# The role of national nutrition programs on stunting reduction in Rwanda using machine learning classifiers: a retrospective study

**DOI:** 10.1186/s40795-024-00903-4

**Published:** 2024-07-11

**Authors:** Jacques Munyemana, Ignace H. Kabano, Bellancile Uzayisenga, Athanase Rusanganwa Cyamweshi, Emmanuel Ndagijimana, Emmanuel Kubana

**Affiliations:** 1 African Centre of Excellence in Data Science, University of Rwanda, Kigali, Rwanda; 2 Rwanda Agriculture and Animal Resources Development Board, Kigali, Rwanda; 3 College of Medicine & Health Sciences, University of Rwanda, Kigali, Rwanda

**Keywords:** Early childhood development, Nutrition sensitive direct support, Antenatal care, Fortified blended food, Stunting reduction, Under-two years, Machine learning, Rwanda

## Abstract

**Background:**

In Rwanda, the prevalence of childhood stunting has slightly decreased over the past five years, from 38% in 2015 to about 33% in 2020. It is evident whether Rwanda's multi-sectorial approach to reducing child stunting is consistent with the available scientific knowledge. The study was to examine the benefits of national nutrition programs on stunting reduction under two years in Rwanda using machine learning classifiers.

**Methods:**

Data from the Rwanda DHS 2015–2020, MEIS and LODA household survey were used. By evaluating the best method for predicting the stunting reduction status of children under two years old, the five machine learning algorithms were modelled: Support Vector Machine, Logistic Regression, K-Near Neighbor, Random Forest, and Decision Tree. The study estimated the hazard ratio for the Cox Proportional Hazard Model and drew the Kaplan–Meier curve to compare the survivor risk of being stunted between program beneficiaries and non-beneficiaries. Logistic regression was used to identify the nutrition programs related to stunting reduction. Precision, recall, F1 score, accuracy, and Area under the Curve (AUC) are the metrics that were used to evaluate each classifier's performance to find the best one.

**Results:**

Based on the provided data, the study revealed that the early childhood development (ECD) program (*p*-value = 0.041), nutrition sensitive direct support (NSDS) program (*p*-value = 0.03), ubudehe category (*p*-value = 0.000), toilet facility (*p*-value = 0.000), antenatal care (ANC) 4 visits (*p*-value = 0.002), fortified blended food (FBF) program (*p*-value = 0.038) and vaccination (*p*-value = 0.04) were found to be significant predictors of stunting reduction among under two children in Rwanda. Additionally, beneficiaries of early childhood development (*p* **< .**0001), nutrition sensitive direct support (*p* = 0.0055), antenatal care (*p* = 0.0343), Fortified Blended Food (*p* = 0.0136) and vaccination (*p* = 0.0355) had a lower risk of stunting than non-beneficiaries. Finally, Random Forest performed better than other classifiers, with precision scores of 83.7%, recall scores of 90.7%, F1 scores of 87.1%, accuracy scores of 83.9%, and AUC scores of 82.4%.

**Conclusion:**

The early childhood development (ECD) program, receiving the nutrition sensitive direct support (NSDS) program, focusing on households with the lowest wealth quintile (ubudehe category), sanitation facilities, visiting health care providers four times, receiving fortified blended food (FBF), and receiving all necessary vaccines are what determine the stunting reduction under two among the 17 districts of Rwanda. Finally, when compared to other models, Random Forest was shown to be the best machine learning (ML) classifier. Random forest is the best classifier for predicting the stunting reduction status of children under two years old.

## Background

Stunting prevalence among children has reduced by 11% worldwide between 2000 and 2020, from 33.1% to 22.0%, because of global efforts to combat chronic malnutrition in newborns and children [1]. Reduced child stunting is the first of six targets in the Global Nutrition Target for 2025 and a crucial indicator of the Second Sustainable Development Goal of Zero Hunger [2].

Over 50% of the annual fatalities of children under the age of five from preventable causes are primarily attributable to malnutrition [3]. Apart from deficiencies in single nutrients, malnutrition is responsible for deaths related to infectious diseases among children under the age of five in developing countries, and it is directly or indirectly to blame for 54% of the 10.8 million deaths per year [4].

The reduction of child stunting has progressed slowly in Asia and Oceania. From 2000 to 2016, stunting decreased drastically in Latin America compared to its reduction in Africa [5]. However, not all demographic groups have experienced the same decline in childhood stunting. For example, between 1990 and 2013, child malnutrition decreased more in urban areas than in rural areas in Asia–Pacific, Latin America, and the Caribbean [6, 7].

Children living in low-income households or whose mothers had a low level of education were more likely to experience stunting. Stunting in children became less common, dropping from 49.3% in 2000 to 39.0% in 2010 [8].

In comparison to other world regions, Sub-Saharan Africa (SSA), which includes Rwanda, has a high prevalence of stunting. Around 42% of children under the age of five are stunted [9]. Infants and pre-school children are the most sensitive to growth retardation caused by malnutrition [10]. From conception to adulthood, enough nutrition is required for proper growth and physical development [11–15].

The most powerful indicators of child stunting are related to the household's socioeconomic situation and environment [16–20]. Child health and not stunting are assumed to be mostly caused by access to clean water, sanitation, and hygiene (WASH) [19–28].

Rwanda is one of the most populous countries in central Africa. According to the 2022 Rwanda Population and Housing Census, the total population is around 13 million, with the majority of the people living in rural areas [29].

Over the past five years in Rwanda, there has been a dramatic change in the national prevalence of child stunting, which is approximately 33%, down from 38% [30]. Since 2015, there have been high changes in 17 districts with a 38% or high prevalence of stunting and high poverty in Rwanda [31]. This shows the heterogeneity in population exposure to the factors that cause child stunting and the need to focus government interventions on the most susceptible group. Additionally, a child undergoes enormous growth and development during the first 1000 days of life [6]. It is commonly known that at least 80% of brain development happens before the age of two, and delays after that age are difficult to reverse [6]. That is why the government of Rwanda has established strategies, including the national plan to eliminate malnutrition to improve the nutritional status of children, which encompasses a multisectoral package of nutrition-sensitive and specific interventions focusing on 17 out of 30 districts of Rwanda, the Stunting Prevention and Reduction Project (SPRP). Most of these strategies consisted of early Childhood Development (ECD) programs focusing on teaching approaches, preparing balanced and nutritious diets, and providing supplement foods such as micronutrient powders [32].

While stunting reduction under two years may have several causes, ultimately government officials and stakeholders want to know, “how national nutrition programs have contributed to the stunting reduction under two years in the targeted 17 districts of Rwanda?”. In this background, with the help of data science techniques and other predicting models, the researcher wants to examine the role of national nutrition programs on stunting reduction under two years in Rwanda using machine learning classifiers.

## Materials and methods

### Study design

This study focused on a retrospective cohort study. In 2016, the Local Administrative Entities Development Agency (LODA) and Ministry of Local Government (MINALOC) of Rwanda established a Monitoring and Evaluation Information System (MEIS) to monitor and follow up on all households receiving social protection programs across the country. In 2019, LODA, in collaboration with districts, carried out a household survey to address issues of targeting effectiveness through a review of the current ubudehe categories in order to streamline the implementation of social protection programs [33].

This study analysed secondary data from the MEIS system developed by LODA and household survey conducted in 2019 by LODA to examine the benefits of national nutrition programs on stunting reduction among under two years’ children.

### Study population and sample size

The study population included all households with ubudehe categories 1 and 2 having at least one child under 2 years of age or pregnant women in 17 Rwandan districts with a high prevalence of stunting and poverty compared to other districts in Rwanda, where the majority of nutrition programs were established. The database of this study contains 92,809 households collected from the integration of two databases, the household survey database and the MEIS database. This household survey was conducted by LODA using administered questionnaire. The questionnaire tool was pre-tested and modified after a pilot study in two districts to ensure the reliability and accuracy of tool, highlight where further training of enumerators is needed. Households with the following criteria were excluded: [1] demographic characteristics of household head( age, sex, education level, wealth category) is missing; [2] lack of information on nutrition and health programs such as nutrition sensitive direct support(NSDS), early childhood development(ECD), antenatal care(ANC) of 4 visits, fortified blended food(FBF), all vaccinations required, Kitchen garden and Toilet facility status; lack of information on nutritional status of child. In total, 8141 including both children and pregnant women met the eligibility criteria and were selected in this study as sample size.

### Data collection procedures

In this study, different data sources were employed to compile information on various study variables at the district level. The Rwanda Demographic and Health Surveys were used to determine the prevalence of stunting and the coverage of areas such as vaccination, antenatal care, and child health that may be related to the evolution of the prevalence of stunting [30, 31]. Variables related to social protection programs will be extracted from the MEIS system developed by LODA. Other remaining needed variables will be extracted from a household survey conducted in 2019 by LODA through para-social workers and youth volunteers trained to collect data using questionnaire deployed in their mobile phones.

The terms and conditions for utilizing the household survey data were agreed upon by the researcher, who was prohibited from making disclosure for the household information provided. LODA Board of Directors has approved procedures for using household survey data where the information supplied by household should be treated as strictly confidential and can only be used for research purpose.

### Ethical considerations

The study was carried out upon receiving approval from the Local Administrative Entities Development Agency (reference number: NC/NF/290/2022) on July 21, 2022, to access household profiling data and nutrition-sensitive direct support data from system (MEIS). To protect the privacy of study participants, all personally identifiable information was removed during data extraction, and completely anonymous identification numbers were generated.

###  Statistical analysis

The analysis consisted of descriptive statistics and inferential statistics. Quantitative and qualitative data were analyzed using STATA 13, R, SAS, and Python as statistical tools used to compute coefficients of estimate, survival analysis, and tabulation and evaluation performance of machine learning classifiers. These approaches were selected because they are able to use data from almost any sort of file to create tabular reports and charts, perform descriptive statistics, and carry out sophisticated statistical analysis.

### Analysis performed

#### Kaplan–meier survival curves

$$\widehat S\left(t_{\left(s\right)}\right)\;=\;\widehat S\left(t_{\left(s-1\right)}\right)x\widehat Pr\left(T\;>\;t_{\left(s\right)}\left|(T\;\geq\;t_{\left(s\right)}\right.\right)$$

$$\widehat S\left(t_{\left(s-1\right)}\right)\;=\;\prod\nolimits_{1=1}^{s-1}\widehat Pr\left(T\;>\;t_{\left(i\right)}\right)\left|\left(T\;\geq\;t_{\left(i\right)}\right)\right.$$

The general equation for a KM surviving probability at a stunted time, ***t***_(***s***)_, is displayed above. Given survival to at least time ***t***_(***s***)_, this formula calculates the probability of surviving past the prior stunted time ***t***_(***s*** − **1**)_ by multiplying it with the conditional probability of surviving past time ***t***_(***s***)_. 

When substituting for the survival probability, the product of all fractions estimates the conditional probabilities for failure times ***Ŝ***(***t***_(***s*** − **1**)_) and earlier, the KM formula can also be stated as a product limit [34].

#### Cox proportional hazard model

To estimate the effects of survival risk-related factors on the stunting status, the cox proportional hazard regression model was utilized. The model is useful in analyzing lifetime data. The continuous random variable (t) in the model represents a person's lifetime, and the vector of explanatory factors associated with (X) indicates the proportional hazard hypothesis.

$$\begin{array}{c}h\left(t,X\right)={h}_{0}\left(t\right){e}^{\,\sum\limits_{i=1}^{p}{\beta }_{i}{X}_{i} }\\ X=\left({X}_{1},{X}_{2},\ldots \ldots \ldots ,{X}_{p}\right)\end{array}$$

According to the cox model formula, the hazard at time (t) is the product of two quantities. The baseline hazard function is *h*_0_ (*t*), the first of these [34].

#### Binary logit model specification

The binary logistic regression was used to estimate the coefficients, and odd ratios were used in this analysis to identify the nutrition programs related to stunting reduction in 17 districts of Rwanda after 5 years.The dependent variable is coded as follows: yes = 1 if a child/pregnant woman is not stunted, no = 0 if a child/pregnant woman is stunted.

The child in the selected household is classified as "stunted" or "non-stunted" based on the dichotomous outcome of the user decision, which characterizes the dependent variable (Y). As a result, a household is classified as “non-stunted” when Yi = 1 or as “stunted” when Yi = 0. For such types of dependent variables, either the probit or logit models are appropriate, depending on personal preferences. This model has also been used in an analysis of ethnic minorities' generational progress in the United Kingdom by examining four labor market outcomes: economic inactivity, unemployment, access to salaried jobs, and self-employment [35]. The binary Logit model was used, and its specifications are as follows:

1 $$logit\left(p\right)=\text{log}(\frac{p}{1-p})={\beta }_{0}+{\beta }_{1}{X}_{1}+{\beta }_{2}{X}_{2}+{\beta }_{3}{X}_{3}+\cdots +{\beta }_{n}{X}_{n}$$

where logit (*p*) is the log of the odds $\left(\frac{p}{1-p}\right)$

This can also be expressed in terms of probability *p*, and the model becomes

2 $$Z\;=\;\beta_0\;+\;\beta_1X_1\;+\;\beta_2X_2\;+\;\beta_3X_3\;+\;\cdots\;+\;\beta_9X_9\;+\;\varepsilon\\$$

Z stands for stunting status, which is the outcome dummy variable that indicates whether the child in the selected household is stunted. In this model, ECD attended (*X*_1_) will be used to estimate the contribution of the ECD program in reducing stunting in Rwanda. The level of poverty in households (*X*_2_) was considered to estimate the effect of poverty on stunting reduction. social protection (*X*_3_) was used to estimate its role in stunting reduction, while access to & use of healthcare (*X*_4_), hygiene & sanitation (*X*_5_), household head age (*X*_6_), household head education (*X*_7_), household head sex (*X*_8_), and kitchen garden (*X*_9_) were used to estimate their contributions to stunting reduction. To capture any measurement error in the stunting reduction, the error term (ε) is appended and left out variables.

3 $$P(Y=1/Z)=\frac{{e}^{{\beta }_{0}+{\beta }_{1}{X}_{1}+{\beta }_{2}{X}_{2}+{\beta }_{3}{X}_{3}+\cdots +{\beta }_{9}{X}_{9}}}{1+{e}^{{\beta }_{0}+{\beta }_{1}{X}_{1}+{\beta }_{2}{X}_{2}+{\beta }_{3}{X}_{3}+\cdots +{\beta }_{9}{X}_{9}}}\text{Or}\ P(Y=1/Z)=\frac{{e}^{Z}}{1+{e}^{Z}}$$

when household is not stunted

4 $$P\left(Y=0/Z\right)=1-\frac{1}{1-{e}^{Z}}$$

when household is stunted.

P denotes the probability of not being stunted 1- P is the probability of being stunted.

#### Other used predicting models

Machine learning algorithms offer effective, model-free solutions to categorize issues. As a result, the performances of various ML algorithms and the statistical classifier were indeed compared. They were selected because most of the variables in the dataset were categorical variables. So, machine learning classifiers helped in evaluating the best method for classification. The ML classifiers that were considered in this study are detailed below.

#### Decision trees

In decision tree learning, a decision tree is utilized as a predictive model to connect observations about an item to judgments about the target value of the item. A data mining induction technique called the decision tree algorithm repeatedly divides a dataset of records into classes according to whether they are all members of the depth-first greedy approach or the breadth-first approach [36].

##### Random forest

Random forest is a classification method that focuses on the "rising" of a group of ordered tree classifiers. To classify a new entity, characteristics of this identity are frequently used, employing each classification tree in the forest. The grown trees are built at random, and each tree offers a categorization (or "vote") for a given class name. The choice is made based on votes cast by most of the forest trees [37].

#### K-nearest neighbors

Of all machine learning algorithms, the K-Nearest Neighbor Algorithm is the most straightforward. It is founded on the idea that similar samples will typically be found close together. Because they keep all the training samples and wait to build a classifier until a new, unlabeled sample must be classified, instance-based classifiers are also known as lazy learners [38].

#### Performance criterion

In the study, various evaluation metrics were used to evaluate the prediction models. The criteria are precision, recall score, F1 score, accuracy and AUC score.

## Results

### Comparison between DHS 2014–2015 and DHS 2019–2020

**Fig. 1:**
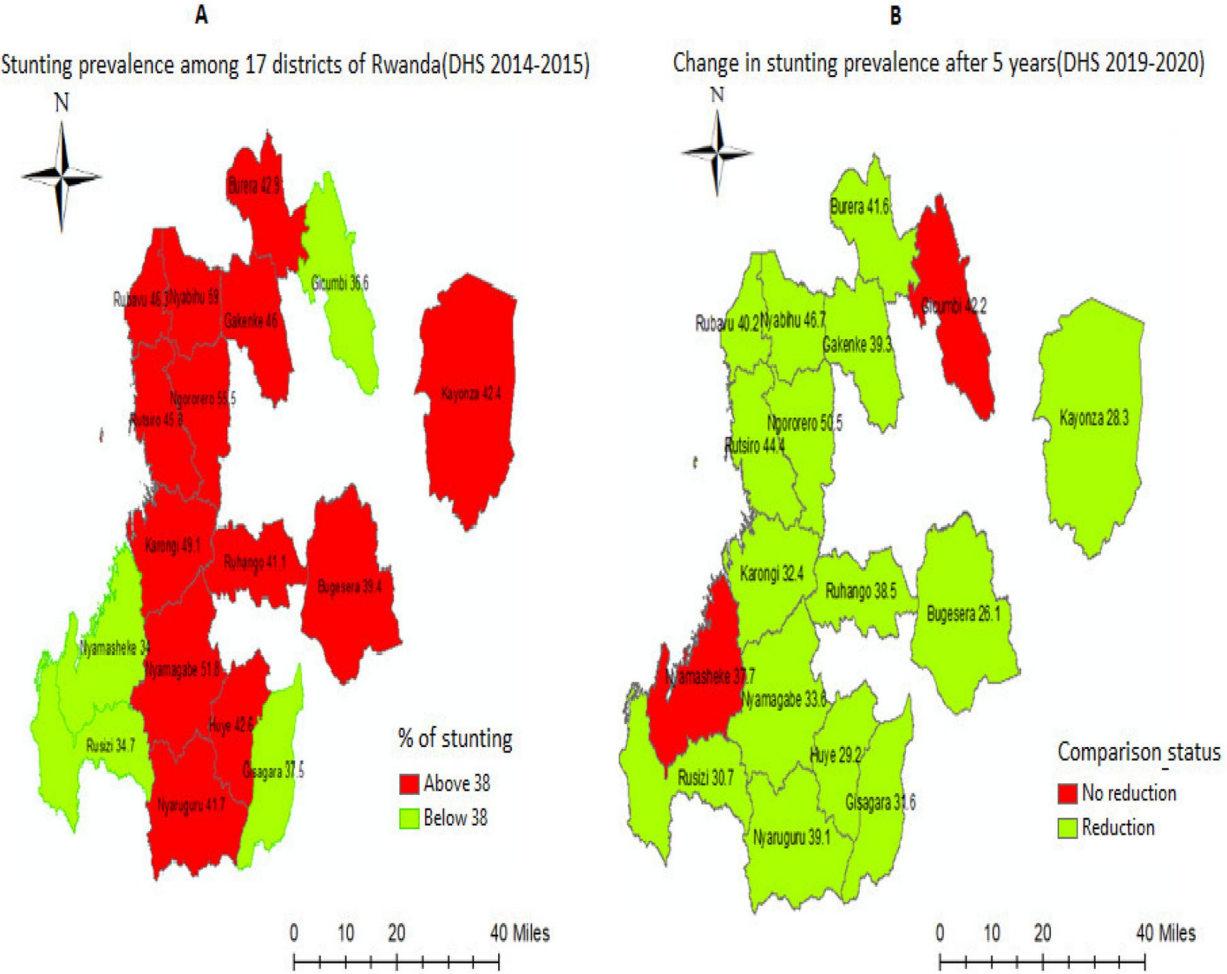
The map of 17 districts of Rwanda

According to the Fig. 1, map A showed that in 2014–2015, 4 districts (green color) consistently had lower stunting prevalence than the national prevalence of stunting in Rwanda (38%) and 13 districts (red color) consistently had greater stunting prevalence. Based on their location and level of poverty, the 4 districts were chosen for the Nutrition-Sensitive Direct Support program (NSDS). Gisagara, Nyamasheke, and Gicumbi districts were picked due to their greater rates of poverty, and Rusizi district was chosen because it is in Rwanda's western region, where most districts had higher rates of stunting than other districts in other regions of the country. The other side of Map B indicated a change in the frequency of stunting after five years (2019–2020). With the exception of Gicumbi and Nyamasheke districts, every other district saw a decrease in stunting from the preceding period (2014–2015). The question is, “How did those districts reduce the stunting prevalence” and “What were the nutrition programs contributing to stunting reduction for the last 5 years?”. Let us look at it by investigating those factors.

### Demographic characteristics of participants

Eight thousand one hundred forty-one pregnant women and children in total participated in the study, with 6268 (77%) of them being children and 1873 (23%) being pregnant women. In addition, 2880 (35.4%) were stunted, whereas 5261 (64.6%) were not.

From a total of 6372 samples that had gone through all four visits necessary for Antenatal Care (ANC), Table 1 shows that 81.14% were stunted-free, whereas the remaining 18.86% were. Antenatal care (ANC) is associated with stunting status (*p*-value = 0.000). 65.12% of the 8070 samples of children who attended early childhood development (ECD) program as a whole were not stunted, compared to 34.88% who were. There is significance of the association between early childhood development (ECD) and stunting status (*p* = 0.000). In contrast to households participating in the fortified blended food (FBF) program, a total sample of 6571 individuals showed that 78.51% were not stunted and 21.49% were. The association between fortified blended food (FBF) and stunting status is significant (*p*-value = 0.000). Children who had a literate household head were less likely to be stunted (62.47%) than those who were (37.53%. The association between household head literacy and stunting status is significant (*p*-value = 0.000). When compared to children who were stunted (35.54%), children from households with a kitchen garden were stunted-free at a rate of 64.46%. There is no significance association between kitchen garden and stunting status(*p*-value = 0.059). Of the households using Mituelle as medical insurance, 65.68% were not stunted, compared to stunted 34. 32%. While the households using other medical insurance, 35% were not stunted compared to 65% stunted. In the households using no medical insurance, 2.4% were not stunted compared to 97.6% stunted. The study proved that there is a significant association between medical insurance and stunting status(*p*-value = 0.000). Compared to households receiving the nutrition sensitive direct support (NSDS) program, 81.55% had no stunted children, whereas 18.45% had. The association between nutrition sensitive direct support (NSDS) and stunting status is significant (*p*-value = 0.000). When comparing sex households headed to being stunted, males (64.25%) were more likely to not be stunted compared to stunted 35.75%. On the other hand, females (67.14%) were more likely to not be stunted compared to stunted 32.86%. There is no significance of the association between household head sex and stunting status (*p* = 0.929). In the households using improved toilets,70% were not stunted compared to stunted (30%). There is significance of the association between toilet facility and stunting status (*p* = 0.000). In the same way, households in category 1 of ubudehe (very poorest) had a higher percentage of not being stunted (78.45%) than stunted households (21.55%). There is significance of the association between ubudehe category(lowest wealth quintile) and stunting status (*p* = 0.000). Children or pregnant women who received all required vaccines were not stunted by 81.45% compared to stunted (18.55%).The association between vaccination and stunting status is significant (*p*-value = 0.000).

**Table 1:** Descriptive statistics of dummy variables for stunting reduction

Covariate	Not stunted		stunted		N	*P*-value
	**n**	**%**	**n**	**%**		
**Antenatal care (ANC) Visits**						
Completed 4 visits	5170	81.14	1202	18.86	6372	0.000
Not completed 4 visits	91	5.14	1678	94.86	1769	
**Household attending early childhood development (ECD)**						
Yes	5255	65.12	2815	34.88	8070	0.000
No	6	8.45	65	91.55	71	
**Household receiving fortified blended food (FBF)**						
Received	5159	78.51	1412	21.49	6571	0.000
Not received	102	6.50	1468	93.50	1570	
**Household head education**						
Literate	3587	62.47	2155	37.53	5742	0.000
Illiterate	1674	69.78	725	30.22	2399	
**Having kitchen garden**						
Yes	5109	64.46	2817	35.54	7926	0.059
No	152	70.70	63	29.30	215	
**Medical insurance**						
Mituelle	5251	65.68	2744	34.32	7995	0.000
Others	7	35.00	13	65.00	20	
None	3	2.38	123	97.62	126	
**Household receiving nutrition sensitive direct support (NSDS)**						
Received	5158	81.55	1167	18.45	6325	0.000
Not received	103	5.67	1713	94.33	1816	
**Sex of Household head**						
Male	4548	64.25	2531	35.75	5879	0.929
Female	713	67.14	349	32.86	2262	
**Toilet facility**						
Improved toilet	5236	70.07	2237	29.93	7473	0.000
Unimproved toitet	25	3.74	643	96.26	668	
**Ubudehe category**						
Category 1	6154	78.45	1691	21.55	7845	0.000
Category 2	151	7.04	145	92.96	296	
**Vaccination program**						
Received all required vaccines	5158	81.45	1175	18.55	6333	0.000
Not received all required vaccines	103	5.70	1705	94.30	1808	

It is important to remember that this section of data analysis employs the chi-square test to determine the relationship between the dependent variable and the covariates (categorical variables only). The level of significance is set at 95%. The *p*-value is less than 5%, indicating the significance of the association between them and the covariate chosen to be used in the final model. The non-statistically significant covariates were not included in the final model.

The Table 2 shows that the average age of the household head is 39.3 for people who are not stunted and 35.8 for those who are stunted, with standard deviations of 14.1 and 11.4, respectively. At the 5% statistical significance level, there is a mean difference in age of household heads between those who are not stunted and those who are stunted. The means of household size for not stunted and stunted are 4.4 and 4.3 with standard deviations of 1.70 and 1.68, respectively. With a 5% difference, the mean household size between those who are not stunted and those who are is statistically significant. Average child ages for not stunted and stunted children are 20.6 and 23.5 months, respectively, with standard deviations of 8.5 and 7.55. This finding demonstrated that there is a mean difference between children who are not stunted and those who are stunted at the age of stunting.

**Table 2:** Descriptive statistics of continuous variables for stunting reduction

Covariate	Not stunted		Stunted		T-test
	Mean	Standard deviation(SD)	Mean	Standard deviation(SD)	
**Age of Household Head**	39.277134	14.060759	35.765278	11.38726	0.000
**Household size**	4.406197	1.703215	4.314236	1.676163	0.02
**Child age(month)**	20.573085	8.586212	23.489931	7.559552	0.000

### Kaplan–meier survival curves analysis

**Fig. 2:**
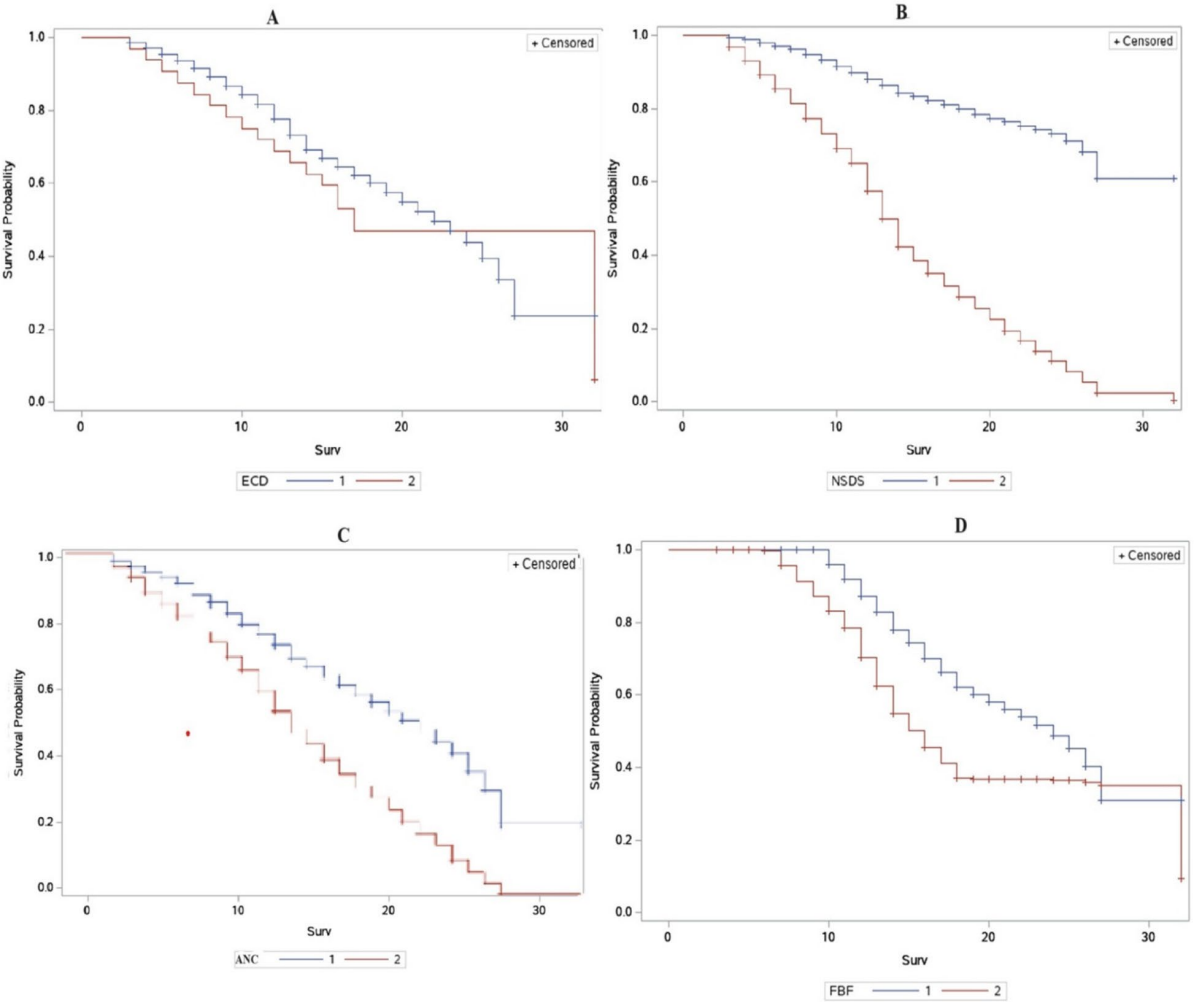
Kaplan–meier curves of selected programs

The kaplan–meier (KM) curves of groups 1 and 2 for four government programs, including the early childhood development, nutrition sensitive direct support, antenatal care, and fortified blended food programs, are shown in the Fig. 2. Here, the survival time was the age of the child, and the event was getting stunted. The two groups differ primarily at larger or smaller survival times. The kaplan–meier (KM) curve for group 1 is continuously greater than the kaplan–meier (KM) curve for group2, according to Figure A, which indicates the early childhood development (ECD) program. According to these data, households participating in early childhood development (ECD) (group 1) had a longer survival rate than households participating in early childhood development (ECD) (group 2) for the first 23 months. Additionally, the two curves seem to diverge as the number of months rises, indicating that the kaplan–meier (KM) curve for group 2 is continuously greater than the kaplan–meier (KM) curve for group 1. In a similar graph, Figure B indicates the nutrition sensitive direct support (NSDS) program and shows that group 1's kaplan–meier (KM) curve is consistently greater than group 2's kaplan–meier (KM) curve. These statistics reflected that households receiving nutrition sensitive direct support (NSDS) (group 1) had greater survival rates than those who did not (group 2). Figure C shows that the women who had visited a health care provider at least 4 times during their pregnancy have a better survival time than those who did not. Finally, Figure D indicates that households that received fortified blended food (FBF) had a better survival time for the first 28 months than those that did not.

### Cox proportional hazard model on the influence of social and economic factors on the prevalence of stunting in children under two years

The Table 3 indicates that the risk of stunting for households that participated in an early childhood development (ECD) program is 0.691 times that of a household that did not participate in an early childhood development (ECD) program. There is a 31% reduction. This demonstrated the program's role in reducing stunting prevalence among children under the age of two. The parameter of having a child stunted-free while attending early childhood development (ECD) in the home is significant (*p*-value = 0.0001). Children who lived in households that received the nutrition sensitive direct support (NSDS) program had a lower risk of stunting than those who did not (HR:0.137, *p*-value:0.0055). Similarly, poor (category 2) households were more likely to have a child stunted than very poor (category 1) households (HR:0.235, *p*-value:0.0146). The risk of a child being stunted decreases as the age of the household head increases (HR:0.989, *p*-value:0.0022). The table indicates that as household size increases, the risk of a child being stunted before the age of two increases (HR:1.029, *p*-value:0.1939). The parameter of household size is not significant. The children of literate household heads had no greater risk of stunting than those of illiterate household heads, and this parameter is not statistically significant (HR:1.005, *p*- value:0.9445). Women who completed at least four visits to health care providers had a lower risk of their child being stunted before the age of two than those who did not. Children who lived in households that received the Fortified Blended Food (FBF) program had a lower risk of stunting than those who did not (HR:0.094, *p*-value:0.0136). This demonstrated the importance of fortified blended food in reducing malnutrition among children under the age of two. Finally, vaccinated children were less likely to be stunted than unvaccinated children (HR:0.586, *p*-value:0.0355).

**Table 3:** Cox proportional hazard model results

Parameter	Parameter estimate	Standard error	Chi – square	*P*-value	Hazard ratio (HR)
**Early Childhood Development** (**ECD) program (ref.Not attended)**					
Attended	1.06171	0.21384	24.6517	** < .0001**	0.691
**Nutrition Sensitive Direct Support** (**NSDS) program (ref. Not received)**					
Received	-1.98526	0.71491	7.7114	**0.0055**	0.137
**Ubudehe category(ref. Category2)**					
Category1	2.32585	0.95210	5.9675	**0.0146**	0.235
**Age Household Head**	-0.01091	0.00356	9.3808	**0.0022**	0.989
**Household size**	0.02858	0.02200	1.6877	0.1939	1.029
**Household head literacy(ref.Illiterate)**					
Literate	0.00508	0.07266	0.0049	0.9443	1.005
**Antenatal Care (ANC) program (ref. Not completed)**					
Completed	-0.87571	0.41370	4.4808	**0.0343**	0.417
**Fortified Blended Food (FBF) program (ref. Not received)**					
Received	-2.36238	0.95729	6.0900	**0.0136**	0.094
**Vaccination program (ref. Not completed)**					
Completed	1.12676	0.58847	3.6662	**0.0355**	0.586

### Nutrition programs associated to stunting reduction in 17 districts of Rwanda after 5 years

The Table 4 shows that the model's outcomes identified the nutrition intervention associated with stunting reduction in 17 districts of Rwanda after 5 years. The following is the interpretation of the findings:

**Table 4:** Logit model results

Variable	Odds Ratio	Standard error	z	P >|z|	[95% Conf. Interval]	
**Early Childhood Development** (**ECD) program (ref. Attended)**						
Not attended	0.406622	0. 324,366	-2.04	**0.041**	0.172037	0.961078
**Nutrition Sensitive Direct Support** (**NSDS) program (ref. Received)**						
Not received	0.463643	0.3558522	-2.16	**0.030**	0.340384	0.946425
**Ubudehe category (ref. Category 1)**						
Category 2	0.3313839	0.4037984	-8.25	**0.000**	0.2550249	0.4310161
**Age Household Head**	0.9997754	0.003855	-0.06	0.954	0.9922482	1.00736
**Household size**	1.029195	0.0285481	1.04	0.300	0.9747357	1.086697
**Household head literacy(ref.Illiterate)**						
Literate	1.110183	0.1044752	1.11	0.267	0.9231904	1.335052
**Medical insurance (ref. Mituelle)**						
None	0.6717147	0. 151,436	-1.77	0.078	0. 4,318,009	1.044928
Others	0.413503	0.2192953	-1.67	0.096	0.1462367	1.169233
**Toilet facility status (ref. Improved)**						
Unimproved	0.3091769	0.0997762	-3.64	**0.000**	0.1642522	0.5819731
**Antenatal Care (ANC) program (ref. Not completed)**						
Completed 4 visits	1.653178	0. 263,289	3.16	**0.002**	1.209916	2.258832
**Fortified Blended Food** (**FBF) program (ref. Not received)**						
Received	1.536144	0. 317,898	2.07	**0.038**	1.023952	2.30454
**Vaccination program (ref.Not vaccinated)**						
Vaccinated(All)	1.527712	0. 1,828,006	2.04	**0.04**	1.018872	2.290676

There is enough evidence from the model output to confirm that the model is correct: the stunting reduction in under two year old children among the 17 districts of Rwanda is determined by attending the early childhood development (ECD) program, receiving the nutrition sensitive direct support (NSDS) program, the focus on households with the highest poverty level (ubudehe category), sanitation facilities (having improved toilets), visit health care providers at least 4 times during the antenatal care (ANC), receiving fortified blended food (FBF), and vaccinating all required vaccines for a specified period.

The odd ratio of 0.4066 [95 percent CI: 0.172–0.961] of children being stunted-free for households attending the ECD program is greater than 59.3 percent of those not attending, indicating that households attending the early childhood development (ECD) program have a greater chance of a child not being stunted than those who do not. This implies that early childhood development (ECD) provides an important window of skills needed by mothers for child’s development. Skills provided by ECD include breastfeeding and feeding practices, how to prepare a balanced diet, how to interact psychologically and environmentally with children under two years old, brain stimulation, and positive parenting. Similarly, the odd ratio of children being stunted-free in households that received nutrition sensitive direct support (NSDS) is more than 53.6 percent higher than in households that did not receive 0.464 [95 percent CI: 0.340–0.946].

This means that households that do not receive an unconditional cash transfer have a lower chance of being stunted-free. The cash transfers provided by the government through nutrition sensitive direct support program help vulnerable families to strengthen their knowledge on better parenting and childcare.

Children who lived in households with a high level of poverty (category 1) have a larger chance of being stunted- free compared to (category 2) 0.331 [95 percent CI: 0.255–0.431]. This indicated the focus of government interventions to reduce malnutrition among children in ubudehe category 1 (the poorest one) by providing them grant in form of cash transfers to vulnerable pregnant women and lactating mothers. Improved toilet facilities were a significant predictor of being stunted-free; the odd ratio for those with good toilet facilities is 0.309 [95 percent CI: 0.1642–0.5819]. This implies that having improved toilet facility can lead to disease prevention and contribute to undernutrition and stunting reduction. Women who made at least four visits to health care providers were 65% more likely to be stunted-free than those who did not 1.653 [95 percent CI:1.2099–2.2588]. This implies that antenatal care (ANC) delivers services to prevent pregnancy complications which can affect low birth weight and continues to affect growth, especially in the first 2 years of life. The odd ratio of stunted-free children in households that received fortified blended food (FBF) is more than 53% higher than in households that did not receive FBF 1.536 [95 percent CI: 1.024–2.304]. This implies that fortified food has impact on nutritional status for pregnant and lactating women. Finally, the odd ratio of children who received all required vaccines was 52.8% higher than those who did not receive all required vaccines: 1.5277 [95 percent CI: 1.0188- 2.29076]. Prevention of diseases preventable by vaccination can lead to undernutrition and stunting reduction.

In this study, the confounding factors of stunting reduction were sex and literacy level of household head. The great number of females headed was confounded with the great number of children stunted. Similarly, the great number of illiterate household heads was confounded with the great number of children stunted. During the study design, the variables were controlled by including female headed with child stunted who is matched to two children not stunted with the same category of female headed. The same way, including illiterate household head with child stunted who is matched to two children not stunted with the same literacy level.

### Performance with other machine learning classifiers

The Table 5 shows that machine learning methods were more effective than binary logistic regression for developing high-model-accuracy stunting reduction prediction models. The F1 score, which is the harmonic mean of precision and recall, accuracy, and the AUC score were used to evaluate the classifier's performance in order to select the best classifier. Among the four classifiers used in this study, the random forest classifier outperformed the other machine learning (ML) classifiers on all performance metrics except precision, where K-nearest neighbors outperformed the other classifiers. This demonstrated that its performance provides a more reliable estimate of feature importance and that it can be used to predict some health indicators in a variety of ways.

**Table 5:** Machine learning classifiers results

Model	Precision score	Recall score	F1 score	Accuracy	AUC score
Logistic regression	0.675958	0.759593	0.715339	0.639589	0.611010
Decision Tree	0.692626	0.787001	0.736804	0.664799	0.635697
Random forest	0.837428	0.907596	0.871101	0.839869	0.823740
K-Nearest Neighbors	0.904934	0.588880	0.713472	0.718021	0.748775

## Discussion of the results

Accelerating the decrease in stunting would require successful, comprehensive nutrition-sensitive programs that target important underlying nutrition-related factors and improve the reach and efficacy of nutrition-specific interventions [39].

This study investigated the nutrition drivers of reducing stunting in under two-year-old children in 17 districts of Rwanda. The findings showed that the main factors influencing the stunting reduction were attending ECD services, receiving nutrition-sensitive direct support (NSDS), supporting vulnerable households of category 1 of ubudehe, having an improved toilet facility, completing at least 4 visits to a health care provider during the pregnancy, receiving fortified blended food (FBF), and vaccinating all required vaccines.

Focusing on household with ubudehe category 1(very poorest) and vaccinating children all required vaccines for have an impact on stunting reduction among under two years’ children. This result is similar with the study conducted on drivers of stunting reduction in the Kyrgyz Republic in 2020, decreasing poverty by focusing on the poorest people and vaccinating some diseases like diphtheria, tetanus, and pertussis has a positive effect on stunting reduction [11].

Household receiving nutrition sensitive direct support (NSDS) has an impact on stunting reduction among under two years’ children. This finding is similar with the result of study investigated the factors that influence stunting reduction in Peru and discovered that children who lived in households that received cash transfer were less likely to be stunted; cash transfers was one of the factors affecting the stunting reduction in Peru [40]. Increased financing of vulnerable households by providing them with social protection support was one of the key drivers of the stunting reduction [40].

Access to toilet facility has an influence on stunting reduction among under two years’ children. This result is similar with the result of study on factors associated with stunting among children aged 0–23 months in Indonesian children was conducted in 2016[41].The study discovered that the combination of improved toilet and treated drinking water was associated with an increased odds-on stunting reduction. Children who live in a house without a toilet were shown to have a significantly higher frequency of stunting than those who reside in a house with a latrine, at 35.3% and 24.0%, respectively [41–43].

Household attending early childhood development (ECD) has an impact on stunting reduction among under two years’ children. This result does not contradict with the finding of study was undertaken by the Integrated Child Development Services (ICDS) Program on a program and intervention for caregivers (knowledge and behavior), family/household (house hygiene and family toilet hygiene), and the community environment (clean water facilities, environmental cleanliness) in 2018. In this study, the intervention group received treatment in the form of education, hygiene promotion, and sanitation. Children in the intervention group had better dental growth than children in the control group (*p* = 0.01). The number of teeth a youngster had correlated with his or her growth [44].

Visiting health care providers at least four times during the pregnancy period has an impact on improvement of nutrition and stunting status among under two years’ children. This result is similar with an Indian study that looked at the relationship between antenatal care and child nutrition improvement in 2022 and found that a set of antenatal interventions, including systems-strengthening strategies and focused nutritional counseling throughout pregnancy, were linked to better nutritional outcomes for children beyond those related to birth [45].

Household receiving fortified blended food (FBF) has an impact on stunting reduction among under two years’ children. This result does not contradict with the finding of research was done on the effect of consumption of micronutrient-fortified foods on the risk of stunting in 2011. It was indicated that children aged 6 to 59 months who consumed micronutrient- fortified milk or micronutrient-fortified noodles had a lower risk of stunting than children who did not consume micronutrient-fortified milk. Stunting risk was lowest in children who drank both micronutrient-fortified milk and micronutrient-fortified noodles[46–49].

Finally, when compared to other models, Random Forest is the best classifier for predicting the stunting reduction status of children under two years old. This result is similar with an employing advanced supervised machine learning approaches for predicting micronutrient intake status among children aged 6–23 months in Ethiopia in 2024. The study found that the random forest, catboost, and light gradient boosting algorithm outperformed in predicting micronutrient intake status among all selected classifiers [50].

### Study limitations

The study was confounded by two factors: sex and literacy level of household head. To control the confounding variables by applying matching may result to selection bias. In addition, the inclusion and exclusion criteria of selecting participants due to the missing data, incomplete data lead to coverage bias. Despite these limitations, the sample size was still large enough required for analysis and the objective of examining the benefits of national nutrition programs on stunting reduction in under two years was achieved.

## Conclusion and recommendation

In general, national nutrition programs hold a lot of promises for improving child stunting, and benefits of nutrition-specific actions. Integrating early childhood development, nutrition sensitive direct support, fortified blended food may result in improvements in reducing child stunting rate. It would be beneficial to concentrate on the crucial window of maximum susceptibility for both nutrition and health during the first 1000 days of life by attending the early childhood development (ECD) program, providing nutrition sensitive direct support and fortified blended food to household with lowest poverty category, having an improved toilet facility to household, vaccinating all required vaccines to child and visiting health care providers at least four times during the pregnancy period. Finally, when compared to other models, Random Forest was shown to be the best machine learning (ML) classifier. This demonstrated that its performance provides a more reliable estimate of feature importance and that it can be used to predict stunting reduction rate.

This study summarized national nutrition programs implemented in various districts to prevent and reduce stunting rates. However, the study did not focus on certain socioeconomic such as marital status, occupation of household head and household residence that may contribute to the reduction of stunting. So, further research to assess the impact of the overall socio-economic factors on stunting reduction were recommended.

## Data Availability

Data was provided in the supplementary information.

## Abbreviations

SSA: Sub-Saharan Africa
NISR: National Institute of Statistics of Rwanda
DHS: Demographic and Health Survey
WHO: World Health Organization
SPRP: Stunting Prevention and Reduction Project
ECD: Early Childhood Development
HR: Hazard Ratio
WASH: Water, Sanitation, and Hygiene
UNICEF: United Nations International Children’s Emergency Fund
MINALOC: Rwanda Ministry of Local Government
LODA: Local Administrative Entities Development Agency
MEIS: Monitoring and Evaluation Information System
NSDS: Nutrition-Sensitive Direct Support
ANC: Antenatal Care
SD: Standard Deviation
KM: Kaplan–Meier
CI: Confidence Interval
FBF: Fortified Blended Food
ML: Machine Learning
AUC: Area Under the ROC Curve
ICDS: Integrated Child Development Services
